# Deceiving Phenotypic Susceptibility Results on a *Klebsiella pneumoniae* Blood Isolate Carrying Plasmid-Mediated AmpC Gene *bla*_DHA-1_

**DOI:** 10.3389/fcimb.2021.561880

**Published:** 2021-03-15

**Authors:** Susan Realegeno, Kevin Ward, Omai B. Garner, Shangxin Yang

**Affiliations:** UCLA Clinical Microbiology Laboratory, Department of Pathology & Laboratory Medicine, University of California, Los Angeles, Los Angeles, CA, United States

**Keywords:** carbapenem resistance, whole-genome sequence analysis, DHA-1, plasmid-mediated AmpC, *Klebsiella pneumoniae*

## Abstract

Carbapenem-resistant *Klebsiella pneumoniae* (CRKP) frequently causes hospital-acquired infections and is associated with high morbidity and mortality. CRKP can have multiple resistance mechanisms and only a few can be routinely detected by commercial molecular or phenotypic assays making surveillance for CRKP particularly challenging. In this report, we identified and characterized an unusual non–carbapenemase-producing CRKP carrying a rare plasmid-borne inducible AmpC gene, *bla_DHA-1_*. The isolate was recovered from blood culture of a 67-year-old female presenting with sepsis post bladder surgery and ureteral stent removal. The primary isolate displayed an indeterminate susceptibility pattern for ceftriaxone by broth microdilution, but was susceptible by disk diffusion with one colony growing within the zone of inhibition. The ceftriaxone resistant colony was sub-cultured and had a minimum inhibitory concentration (MIC) of 2 ug/ml for imipenem (intermediate) and a zone size of 18 mm for ertapenem (resistant), but remained susceptible to cefepime and meropenem. Further phenotypic characterization of this sub-cultured isolate showed carbapenemase activity. Whole genome sequencing (WGS) revealed the presence of two subpopulations of a *K. pneumoniae* (MLST sequence type 11) from the primary blood culture isolate: one pan-susceptible to beta-lactams tested and the other resistant to the 3^rd^ generation cephalosporins and ertapenem. WGS analysis identified the resistant *K. pneumoniae* harboring IncFIB(K) and IncR plasmids and the presence of plasmid-borne beta-lactam resistance genes *bla*_OXA-1_ and *bla*_DHA-1_, an inducible AmpC gene. Additional resistance genes against quinolones (*aac(6′)-Ib-cr, oqxA, oqB*), aminoglycoside (*aph(3′)-Ia*), sulfonamide (*sul1*), and tetracycline (*tet*(A)) were also identified. DHA-1 positive *K. pneumoniae* have been previously identified outside the US, particularly in Asia and Europe, but limited cases have been reported in the United States and may be underrecognized. Our study highlights the importance of using both extended phenotypic testing and WGS to identify emerging resistance mechanisms in clinical Enterobacterales isolates with unusual antimicrobial resistance patterns.

## Introduction

*Klebsiella pneumoniae* is a gram-negative rod and a member of the Enterobacterales family. These organisms are known to cause significant nosocomial infections with a wide range of clinical presentations including pneumonia, bacteremia, and urinary tract infections. One of the most concerning aspects of infection with *K. pneumoniae* is the high prevalence of drug resistance that can limit treatment options. Beta-lactamases are one of the most significant mechanisms of resistance in *K. pneumoniae*, including extended spectrum beta-lactamase (ESBLs) and carbapenemases, which are capable of hydrolyzing penicillins, cephalosporins, and carbapenems. From 1998 to 2010, *K. pneumoniae* surveillance isolates in the United States (US) showed a significant increase in antimicrobial resistance to drugs of all classes, except tetracyclines (Sanchez et al., 2013).

One under-recognized resistance mechanism of particular concern is plasmid encoded AmpC-type beta-lactamase. AmpC type beta-lactamases are part of the Ambler class C group of beta-lactamases that display resistance to penicillins, first, second, and third generation cephalosporins, cephamycins, and monobactams but are not susceptible to commonly used beta-lactamase inhibitors such as clavulanate, sulbactam, and tazobactam (Jacoby, 2009). Inducible AmpC beta-lactamase activity is typically chromosomally encoded and is characteristic in a group of Enterobacterales species, commonly referred to as the “SPICE” group, which include *Serratia marcescens*, *Pseudomonas aeruginosa*, indole-positive *Proteus*, *Citrobacter freundii*, and *Enterobacter cloacae*. *Klebsiella* species do not have chromosomally encoded AmpC, but can acquire the resistance gene through plasmids. Plasmid-borne AmpC gene usually lacks genetic components that regulate AmpC expression and is therefore frequently found to be constitutively expressed. One exception is plasmid encoded *bla_DHA-1_* which is usually adjacent to *ampR*, the transcriptional regulator gene for activation or repression of AmpC (Barnaud et al., 1998; Verdet et al., 2006; Compain et al., 2014; Luan et al., 2015), making it similar to inducible chromosomal AmpC enzymes.

In this report, we identified a clinical carbapenem-resistant *Klebsiella pneumoniae* (CRKP) isolate with inducible *bla_DHA-1_* AmpC in a mixed bacterial population that initially showed inconsistent and confusing phenotypic susceptibility results which prompted further investigation. There are currently no established guidelines for the detection of plasmid-mediated AmpC expression in the clinical microbiology laboratory. WGS was used to identify resistance genes in this isolate, demonstrating that conventional methods are limited in detection of this type of resistance mechanism, which may lead to a vast under-recognition of its prevalence in the community (Jacoby, 2009).

## Materials and Methods

### Antimicrobial Susceptibility and Molecular Testing

A *K. pneumoniae* isolate was recovered from a positive aerobic blood culture bottle and identified by matrix-assisted laser desorption/ionization time-of-flight (MALDI-TOF) using the Vitek MS (BioMerieux, Marcy l’Etoile, France). Initial antimicrobial susceptibility testing was performed using in-house prepared broth microdilution (BMD) trays according to the CLSI guidelines (M07 and M100 29^th^ edition, 2019). Disk diffusion (DD), modified carbapenem inactivation method (mCIM), and modified Hodge-test (MHT) were also performed to further characterize phenotypic resistance mechanisms (CLSI M100 29^th^ edition, 2019 & M07, 11^th^ edition, 2018). A total of 3 isolates are described: the primary isolate (Isolate 1^0^) initially recovered from the blood culture and two isolates representing subpopulations of Isolate 1^0^ separated based on the DD method using a ceftriaxone disk: Isolate 1A was susceptible and Isolate 1B grew inside the inhibition zone. The Xpert CarbaR (Cephid, Sunnyvale, CA) was also performed for detection of specific carbapenemase genes including KPC, NDM, VIM, IMP, and OXA-48-group genes.

### Whole Genome Sequencing

DNA was extracted from bacterial isolates using the Qiagen EZ1 tissue kit (Qiagen, Hilden, Germany) extraction method according to manufacturer’s instructions. Sequencing libraries were prepared using the Illumina DNA Flex kit (Illumina, San Diego, CA) and sequencing was performed on the Illumina MiSeq instrument (Illumina, San Diego, CA) using 2 x 250 protocol. Genomic analysis was done using the KmerFinder, ResFinder, Multi Locus Sequence Typing (MLST), and PlasmidFinder tools provided by the Center for Genomic Epidemiology (http://www.genomicepidemiology.org/). Additional analyses of sequence data were performed using CLC Genomics Workbench v12.0.3 (Qiagen, Hilden, Germany) and Geneious Prime software (Biomatters, Auckland, New Zealand), including mapping, *de novo* assembly, and variant analysis. Sequence data was mapped to the following references: *Klebsiella pneumoniae* strain KP38731, complete genome (Genbank NZ_CP014294.1), and *Klebsiella pneumoniae* plasmid pKPS30, complete sequence (Genbank NC_023314.1).

## Results

### Patient History and Antibiotic Susceptibility Results

**Figure 1:**
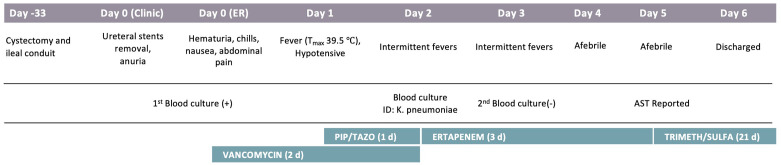
Patient Clinical Course. Significant events are noted under each hospitalization day, including procedures, symptoms, and antibiotics course. AST, Antimicrobial susceptibility testing.

A 67-year-old woman with a history of rectal cancer and recently diagnosed bladder cancer experiencing anuria presented to the clinic for ureteral stent removal approximately 1 month post bladder surgery, cystectomy and ileal conduit (**Figure 1**). She was referred to the ER for evaluation and was admitted due to hematuria, chills, nausea, abdominal pain and severe sepsis on the same day. A primary bacterial culture (Isolate 1^0^) was recovered from the aerobic blood culture bottle and identified as *Klebsiella pneumoniae* by MALDI-TOF. Antimicrobial susceptibility testing was performed using BMD but wells for ceftriaxone and ceftolozane-tazobactam displayed an indeterminate growth pattern in which the MIC could not be interpreted accurately. Upon repeat testing by DD, a single colony (Isolate 1B) was noted within the zone of inhibition for ceftriaxone and was sub-cultured for further workup.

Further phenotypic testing revealed two sub-populations in the primary culture, one susceptible to ceftriaxone and all other 3^rd^ generation cephalosporins (Isolate 1A) and the other resistant to most cephalosporins and ertapenem (Isolate 1B) (**Table 1**). Isolate 1A showed pan susceptibility to all antibiotics tested except fluoroquinolones. Isolate 1B was resistant to most beta-lactams tested, except ceftazidime-avibactam, cefepime, and meropenem. The contrasting differences in beta-lactam susceptibility results between the two sub-populations in the primary culture prompted us to perform carbapenemase assays and WGS for further investigation.

**Table 1:** Antimicrobial susceptibility testing results.

Antibiotic Class	Antibiotic	Isolate 1^0^				Isolate 1A				Isolate 1B			
		BMD		DD		BMD		DD		BMD		DD	
Beta-lactam	Ampicillin-Sulbactam					8	S			>32	R		
	Amoxicillin-Clavulanate					4	S			>32	R		
	Piperacillin-Tazobactam	64	I	18	I	≤8	S	25	S	>128	R	6	R
	Cefazolin	>32	R	6	R	2	S	23	S	>32	R	6	R
	Cefoxitin			6	R			19	S			6	R
	Ceftriaxone	Indeterminate		25	S	≤1	S	30	S	64	R	10	R
	Cefotaxime			24	S			31	S			6	R
	Ceftazidime	16	R	18	I	≤0.5	S	28	S	>32	R	6	R
	Ceftazidime-Avibactam	≤2	S			≤2	S			≤ 2	S		
	Ceftolozane-Tazobactam	≤0.5	S			≤0.5	S			>32	R		
	Cefepime	≤0.5	S	26	S	≤0.5	S	32	S	≤ 0.5	S	24	SDD
	Aztreonam					≤0.5	S			>32	R		
	Ertapenem	≤0.25	S	20	I	≤0.25	S	30	S	0.5	S	18	R
	Imipenem	1	S	24	S	≤0.25	S	26	S	2	I	26	S
	Meropenem	≤0.25	S	25	S	≤0.25	S	29	S	≤0.25	S	26	S
	Meropenem-Vabrobactam					≤0.6	S			≤0.6	S		
Aminoglycosides	Amikacin	≤4	S	23	S	≤4	S	26	S	≤4	S	23	S
	Gentamicin	≤1	S	22	S	≤1	S	26	S	1	S	23	S
	Tobramycin	4	S	25	S	≤1	S	24	S	4	S	14	I
	Streptomycin						S				S		
Macrolides	Azithromycin					16	S			>32	R		
Tetracyclines	Minocycline					2	S			8	I		
	Tigecycline					0.5				1			
Fluroquinolones	Ciprofloxacin	>4	R	6	R	>4	R	6	R	>4	R	6	R
	Levofloxacin	>4	R			>8	R			>8	R		
	Moxifloxacin					>8				>8			
Polymixins	Colistin	≤2	WT			≤2	WT			≤2	WT		
Folate Pathway antagonists	Trimethaprim/Sulfamethoazole	≤1/20	S	12	I	≤1/20	S	14	I	≤1/20	S	6	R

Broth microdilution (BMD) MIC reported in µg/ml; Disk Diffusion (DD) zone size reported in mm; R, Resistant; I, Intermediate; S, Sensitive, SDD, Susceptible-dose dependent.

### Phenotypic Characterization of Resistance Mechanisms

**Figure 2:**
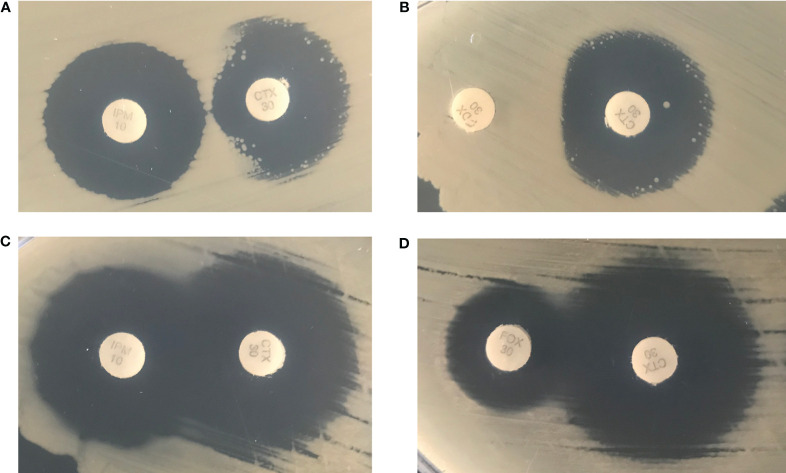
Isolate 1B was cultured in the presence of an imipenem disk (left) placed adjacent to a cefotaxime disk (right) **(A)** and a cefoxitin disk (left) placed adjacent to a cefotaxime disk (right) **(B)**. Isolate 1A was cultured in the presence of an imipenem disk (left) placed adjacent to a cefotaxime disk (right) **(C)** and a cefoxitin disk (left) placed adjacent to a cefotaxime disk (right) **(D)**.

Carbapenemase was tested by MHT, mCIM, and CarbaR PCR assays. No carbapenemase activity was detected in any isolate by MHT. However, Isolate 1B was mCIM positive, while Isolate 1A was negative. CarbaR PCR test, which detects KPC, NDM, VIM, OXA-48-group and IMP genes, was negative in all isolates. Further phenotypic testing was performed to determine possible AmpC activity with cefotaxime adjacent to imipenem or cefoxitin disks on Isolate 1A and 1B (**Figure 2**). Cefoxitin and imipenem were both able to induce resistance against cefotaxime in Isolate 1B but not in Isolate 1A as noted by truncation of the zone, suggesting a possible inducible AmpC resistance mechanism.

### Genetic Determinants of Resistance

Whole genome sequencing was performed on the two sub-populations and the primary mixed culture for comparison to determine genetic relatedness and to identify drug resistance genetic elements. All isolates were identified as Sequence Type (ST) 11 based on MLST analysis and all closely related to the same strain *Klebsiella pneumoniae* strain KP38731 by KmerFinder analysis. Variant analysis was performed using KP38731 as the reference genome and showed no single nucleotide polymorphisms (SNPs) among the four isolates, indicating they are of the same bacterial lineage. Drug resistance genetic analysis using ResFinder identified numerous genes conferring resistance to beta-lactams, aminoglycosides, fluroquinolones, rifamycins, tetracycline, phenicol, macrolides, and fosfomycin in Isolate 1^0^, 1B (**Table 2**). In contrast, 1A had much fewer resistance genes identified (**Table 2**), with only *bla_SHV-182_*, *oqxA*, *oqB*, and *fosA*, which are known to be chromosomally encoded in *K. pneumoniae* (Fu et al., 2007; Ito et al., 2017). In addition, *in silico* plasmid identification analysis by PlasmidFinder detected the presence of two plasmid types, IncFIB(K) and IncR in primary Isolate 1^0^ and Isolate 1B. The InR-type plasmid was not detected in Isolate1A, which was the isolate that demonstrated susceptibility to most antibiotics, further supporting that the primary culture was mixed with two-subpopulations.

**Table 2:** Resistance genes identified by WGS.

Antibiotic Class Targeted	AMR Gene	Isolate 1^0^	Isolate 1A	Isolate 1B
Beta-lactam	blaDHA-1	✓		✓
	blaOXA-1	✓		✓
	blaSHV-182	✓	✓	✓
Aminoglycoside	aac(6*′)*)-Ib-cr	✓		✓
	aph(3*′)*)-Ia	✓		✓
Folate Pathway	sul1	✓		✓
Quinolone	aac(6*′)*)-Ib-cr	✓		✓
	oqxA	✓	✓	✓
	oqxB	✓	✓	✓
	qnrB4	✓		✓
Rifamycins	arr-3	✓		✓
Tetracycline	tet(A)	✓		✓
Phenicol	catB3	✓		✓
Macrolide	mph(A)	✓		✓
Fosfomycin	fosA	✓	✓	✓
**Plasmids**	IncFIB(K)	✓	✓	✓
	IncR	✓		✓

Further BLAST analysis identified the InR-plasmid in Isolate 1^0^ and 1B to be closely related to a previously published InR pKPS30 plasmid (NC_023314.1) with 98.3% pairwise identity and 100% coverage. This 61,288-bp plasmid was initially described in a *K. pneumoniae* ST11 strain isolated in France (Compain et al., 2014). It carries several mobile genetic elements (integron and transposons) with multiple resistance genes, including *bla_DHA-1_, bla_OXA-30,_ aac(6′))-Ib-cr, aphA1, arr-3, catB3, mph(A), qnrB4, aac(6′))-Ib-cr, sul1*, and *tet(A)*, all of which were also identified in Isolate 1^0^ and 1B. Notably, both *bla_DHA-1_* and *ampR*, along with *sul1*, are within a class-1 integron that was originally discovered in a DHA-1-Producing *Klebsiella* spp. in France over 15 years ago (Verdet et al., 2006).

## Discussion

In this report, we describe the detection of AmpC expression in a subpopulation of *K. pneumoniae* recovered from a blood culture using phenotypic antimicrobial susceptibility testing, carbapenemase assays and WGS. From the primary culture isolate, we uncovered two subpopulations: one was ceftriaxone resistant with inducible AmpC and the other was ceftriaxone susceptible without AmpC. The AmpC encoding gene, *bla_DHA-1_*, was detected in Isolate 1B but not in 1A, which is consistent with phenotypic susceptibility results and carbapnemase test results. No carbapenemase encoding genes were detected, suggesting the elevated ertapenem & imipenem MIC and positive mCIM might be due to the hyperproduction of AmpC after induction by cephalosporins or carbapenems.

The patient was admitted due to sepsis-like symptoms and was empirically treated with vancomycin for 2 days and piperacillin-tazobactam for 1 day, both of which would not be effective in treating the *K. pneumonia* isolate. The antibiotic regime was quickly switched to ertapenem on Hospital Day 2 pending susceptibility results, blood cultures were negative by Hospital Day 3, even though the final susceptibility results (reported on Hospital Day 5) showed the bacteria were not susceptible to ertapenem. The short duration of sepsis-like symptoms and suboptimal drug treatment suggested transient bacteremia post ureteral sent removal procedure and antibiotic regime would likely not change the clinical outcome in this case. However, in the context of more severe infection, treatment options are expected to be more challenging due to the limited selection of susceptible beta-lactams, such as imipenem, meropenem, meropenem-vaborbactam, and ceftazidime-avibactam.

Chromosomally encoded AmpC-type beta-lactamases are known to be readily induced by cephamycins and carbapenems (Jacoby, 2009). Non-SPICE group organisms, such as *Escherichia coli* and *K. pneumoniae*, can obtain plasmid-mediated AmpC resistance that is constitutively expressed due to the lack of regulatory genes. However, there is an emerging threat of organisms carrying plasmid mediated inducible AmpC, such as *bla*_DHA-1,_ as identified in this report. Plasmid-mediated *bla_DHA-1_* was first identified in 1992 from a stool isolate of *Salmonella enterica* serovar Enteritidis in Saudi Arabi (Gaillot et al., 1997). Two subsequent DHA-1 producing *K. pneumoniae* isolates were identified in California and Florida (Moland et al., 2002; Alvarez et al., 2004) the same year and additional isolates were later identified in France in 1998 (Verdet et al., 2006). The DHA-1 carrying plasmid in this study is closely related with the plasmid pKPS30 first identified in a ST11 type *K. pneumoniae* in 2008 in a urine isolate from a patient in France (Compain et al., 2014). Plasmid KPS30 contains a 12,391-bp backbone with an IncR replicon and a 44,944-bp MDR region including *bla*_DHA-1_ and *ampR*, insertion sequences, complete class 1 integron, and several transposons. *K. pneumoniae* carrying DHA-1 AmpC have been increasingly reported worldwide, particularly in Europe and Asia (Yan et al., 2002; Lee et al., 2006; Song et al., 2006; Verdet et al., 2006; Vanwynsberghe et al., 2009; Hennequin et al., 2012; Luan et al., 2015) but rarely in the US. In a recent study examining 482 ceftriaxone not susceptible in Enterobacterales isolates in a US medical center, 17% of isolates were found to have a plasmid mediated AmpC and 5% of those were *bla*_DHA_ positive, including one *K. pneumoniae* isolate (Tamma et al., 2019). Although there have been few documented instances of *bla_DHA-1_* carrying *K. pneumoniae* in the US, there are no routine testing methods that can detect this gene and therefore the actual prevalence may be underestimated. Importantly, we demonstrated cefoxitin and imipenem were able to induce resistance to cefotaxime in an isolate harboring the plasmid-borne *bla_DHA-1_*. This would have been concerning if the patient continued to be treated with ertapenem due to the potential for AmpC derepression and therefore carbapenem resistance.

Interestingly, the mCIM test was able to demonstrate carbapenemase activity in our isolate. The mCIM test generally has high sensitivity and specificity for carpabenemase producing organisms but does not typically detect carbapenemase activity in non-carbapenemase producing organisms (Pierce et al., 2017; Zhou et al., 2018; Thomson et al., 2019). Previous studies have shown few positive mCIM results in non-cabapenemase producing organisms, including AmpC producing *Enterobacter* spp. (Pierce et al., 2017; Thomson et al., 2019). Here we demonstrate a *bla_DHA-1_* encoding *K. pneuominae* isolate as another instance of a non–carbapenemase-producing organisms that could result in a positive mCIM. The MHT in all isolates were negative, which contrasts previous studies reporting weakly false positive MHT results in non-carbapenemase, plasmid mediated *bla_DHA-1_* encoded AmpC in *Enterobacter* spp. and *K. pneumoniae* isolates with mCIM negative results (Zhou et al., 2018). In this case, hyperproduction of AmpC is likely causing the hydrolysis of carbapenem which resulted in positive mCIM. According to the current Council of State and Territorial Epidemiologists (CSTE) position statement (Centers for Disease Control and Prevention, 2018), this isolate qualifies as a CRE based on the mCIM positive result alone or the ertapenem resistance seen by disk diffusion. Identification of a CRE is critical for infection control precautions and accurate antibiotic susceptibility testing is important for determining CRE status. However, our study has shown that it is increasingly challenging to detect emerging resistance mechanisms due to limitations of current methods used by many clinical microbiology laboratories.

In summary, we described a case of unusual *K. pneumoniae* bacteremia with a mixed subpopulations of bacteria with or without AmpC gene *bla_DHA-1_*, causing an initial false and discrepant susceptibility profile. Performing disk diffusion to resolve the ceftriaxone susceptibility result was the key in revealing the hidden bacterial subpopulation expressing AmpC. We used WGS to show the mix of bacterial populations were all genetically related except for the presence of an additional plasmid carrying *bla_DHA-1_*. This case highlights the need for guidelines that include both molecular testing and phenotypic screening for inducible AmpC producing organisms outside the typical “SPICE” group. Although plasmid-borne *bla_DHA-1_* is seldom reported in the US, it is possible that organisms with this gene are under detected due to the lack of effective screening methods. WGS is instrumental in revealing inducible resistance mechanisms that are difficult to identify by routine methods. Further molecular epidemiological investigation is required to fully understand the true prevalence of this resistance mechanism in the community.

## Data Availability Statement

Whole genome sequence data presented in this study has been deposited at DDBJ/ENA/GenBank under the accession numbers JADGMK000000000 and JADGMJ000000000.

## Ethics Statement

Ethical review and approval were not required for the study on human participants in accordance with the local legislation and institutional requirements. Written informed consent for participation was not required for this study in accordance with the national legislation and the institutional requirements.

## Author Contributions

SR, KW, and SY participated in data collection, analysis, interpretation, and investigational design. SR wrote the manuscript. All authors reviewed the manuscripts for edits. SY provided project management. OG provided funding, resources, and oversight. All authors contributed to the article and approved the submitted version.

## Funding

This study is funded by the UCLA Department of Pathology and Laboratory Medicine.

## Conflict of Interest 

The authors declare that the research was conducted in the absence of any commercial or financial relationships that could be construed as a potential conflict of interest.
